# Supplementary material for: Flexible categorization in perceptual decision making
Source: Nat Commun. 2021 Feb 24;12:1283. doi: 10.1038/s41467-021-21501-z (PMC7904789; doi:10.1038/s41467-021-21501-z)
Supplement: Supplementary file 1 — Supplementary Information [file 41467_2021_21501_MOESM1_ESM.pdf]

Flexible categorization in perceptual decision making

Genís Prat-Ortega, Klaus Wimmer, Alex Roxin and Jaime de la Rocha

Supplementary information

Supplementary figures pag. 1
Supplementary methods pag. 11

Supplementary figures

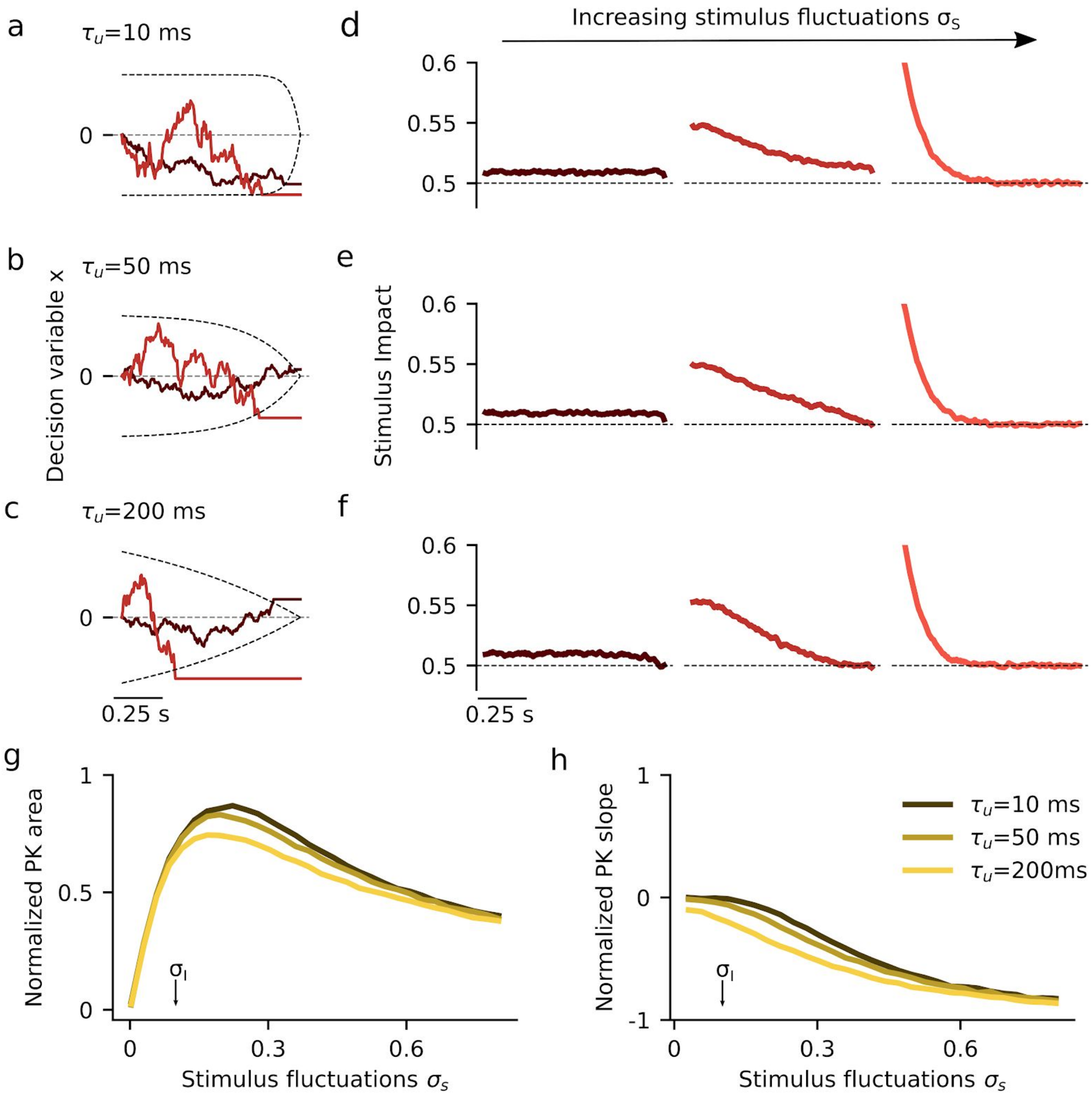

Supplementary Figure 1 | Dynamics of evidence accumulation in the drift diffusion model with collapsing absorbing bounds.

Here we studied a drift diffusion model with collapsing absorbing bounds in which the decision bounds change during the stimulus following $B(t) = B_0 \left(1 - \frac{1-exp(t/\tau_u)}{1-exp(T/\tau_u)}\right)$, where B_0 is the initial bound and T is the stimulus duration. The time during the stimulus in which the collapse is noticeable is set by the time constant τ_u. (**a-c**) Single-trial example traces of the decision variable *x(t)* for weak (brown traces, σ_S=0.03) and intermediate (red traces, σ_S=0.28) stimulus

fluctuations for three collapsing bound time constants τ_u=10, 50 and 200 ms (**d-f**) Psychophysical Kernels (PK) for the same collapsing bound time constants as in (a-c), with increasing magnitude of the stimulus fluctuations (from left to right): σ_S=0.03, 0.28 and 0.69. (**g-h**) Normalized PK area and normalized PK slope as a function of σ_S for the same collapsing bound time constants as in (a-c) (see inset in h). The area is normalized by the PK area of the perfect integrator with no internal noise (σ_i=0) and hence measures the ability of each model to integrate the stimulus fluctuations. Faster collapsing bounds cause a decrease of the PK slope because the bounds are reached earlier during the stimulus presentation. As a consequence the integration becomes more transient decreasing the PK area. In all panels, internal noise was fixed at σ_I=0.1 (see arrows in g and h). Mean stimulus evidence was μ=0 in all cases.

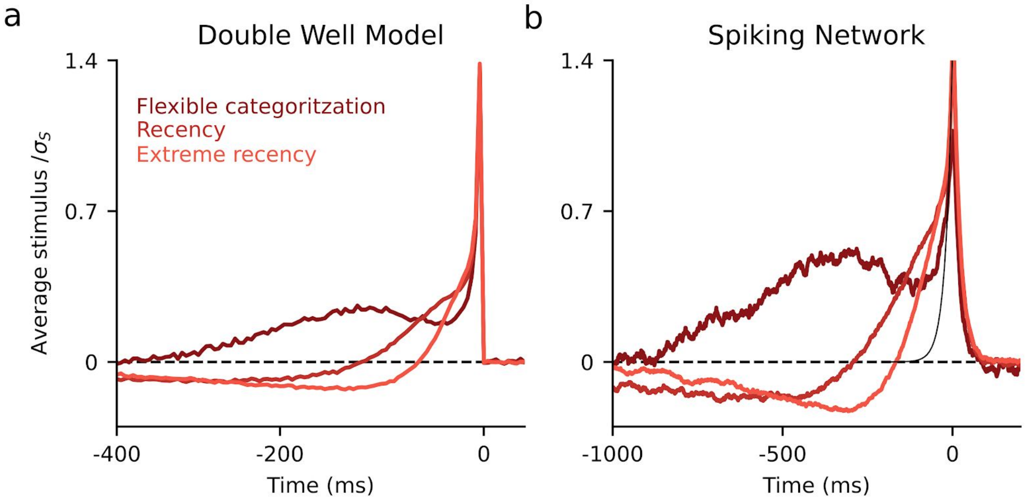

Supplementary Figure 2 | Stimulus integration during a transition between attractor states.

(a) Average stimulus aligned to the transition time (t = 0 ms) in the flexible categorization and recency regime for the DWM (σ_S = 0.5, 1 and 1.5) We introduced two thresholds at the attractor states ($x^*=\pm\sqrt{c_2}/2$) and we defined a transition when the decision variable reached one of the thresholds when the opposite had been previously reached. The peak at t=0 is an artifact produced by crossing the threshold (i.e the last fluctuations was always in favour to cross the threshold). In the flexible categorization regime, the transitions occur when the stimulus favours them for hundreds of ms, considerably longer than the time constant of the system ($\tau = 200\ ms$). Thus the integration of the stimulus continues even when an attractor state has been reached. As stimulus fluctuations increase, the transitions become faster and the system moves into a regime where the transitions are based on momentary evidence rather than an integration of the stimulus (extreme recency). **(b)** Same as in (a) for the spiking network with σ_S=4, 9 and 13, and thresholds at r_A-r_B=±25Hz where r_A and r_B are the firing rates of the two excitatory populations. The stimulus was taken as the difference of the input to populations A and B, $I^A_{stim}(t) - I^B_{stim}(t)$ and μ=0. While the dynamics of the DWM is governed by a single time constant τ , in the spiking network several time constants contribute to the dynamics (Table 2; membrane time constants τ^E_m = 20ms, τ^I_m = 10ms, synaptic time constants τ^E_S =12.5 ms, τ^I_S =1 ms, synaptic delays). Moreover, the stimulus fluctuations are not white noise but correlated noise, realized as an Ornstein-Uhlenbeck process with τ_{stim}=20 ms. The expected decay of the average stimulus is given by the stimulus autocorrelation ($\sim e^{-t/\tau_{stim}}$; black line). Despite all these factors, the transition-triggered average stimuli are qualitatively similar to the DWM. In particular, the longest integration occurs in the flexible categorization regime and exceeds by far all the intrinsic time constants of the network.

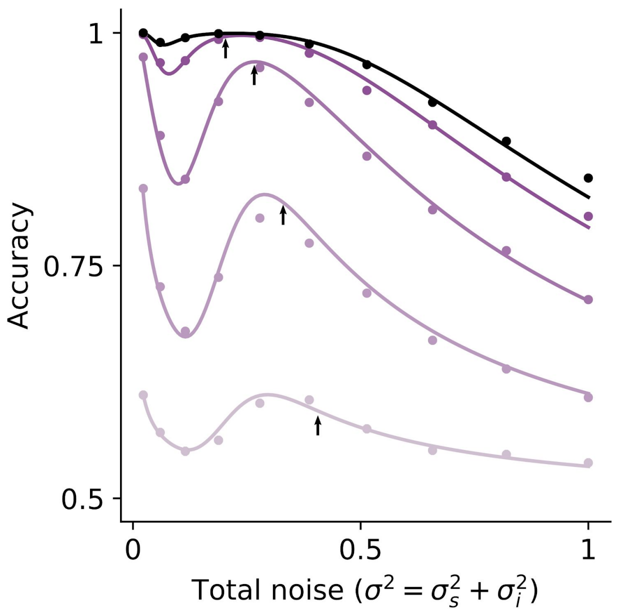

Supplementary Figure 3 | Critical internal noise and mean stimulus evidence compatible with the non-monotonic relation between accuracy and stimulus fluctuations.

Accuracy versus the total noise, $\sigma^2 = \sigma_s^2 + \sigma_i^2$ obtained from simulations (dots) and theory (equation 19, solid line) for different mean stimulus evidence $\mu = 0.03$, 0.1 , 0.2 and 0.3 from light to dark purple as well as the for the critical value $\mu_C = 0.35$ in black. The bump in accuracy occurs if the probability of a correcting transition given by the second term in equation 19 is large enough when the error transition are not activated $(1 - p_E \simeq 1)$. In other words, the accuracy decrease monotonically with σ^2 if in the limited regime where there is a large asymmetry between correcting (p_C) and error (p_E) transitions (Figure 3b), the probability of an error initial categorization (1-P_0) is small and the number of correcting transitions is negligible. Note that the bump becomes smaller when $\mu \to 0$. Thus, intermediate values of mean stimulus evidence are recommended to experimentally test this non-monotonic relation. The black arrows indicate the critical value of the internal noise for a non-monotonic relation between the accuracy and the stimulus fluctuations from equation 40. It is precisely the value of the local maximum in the total noise.

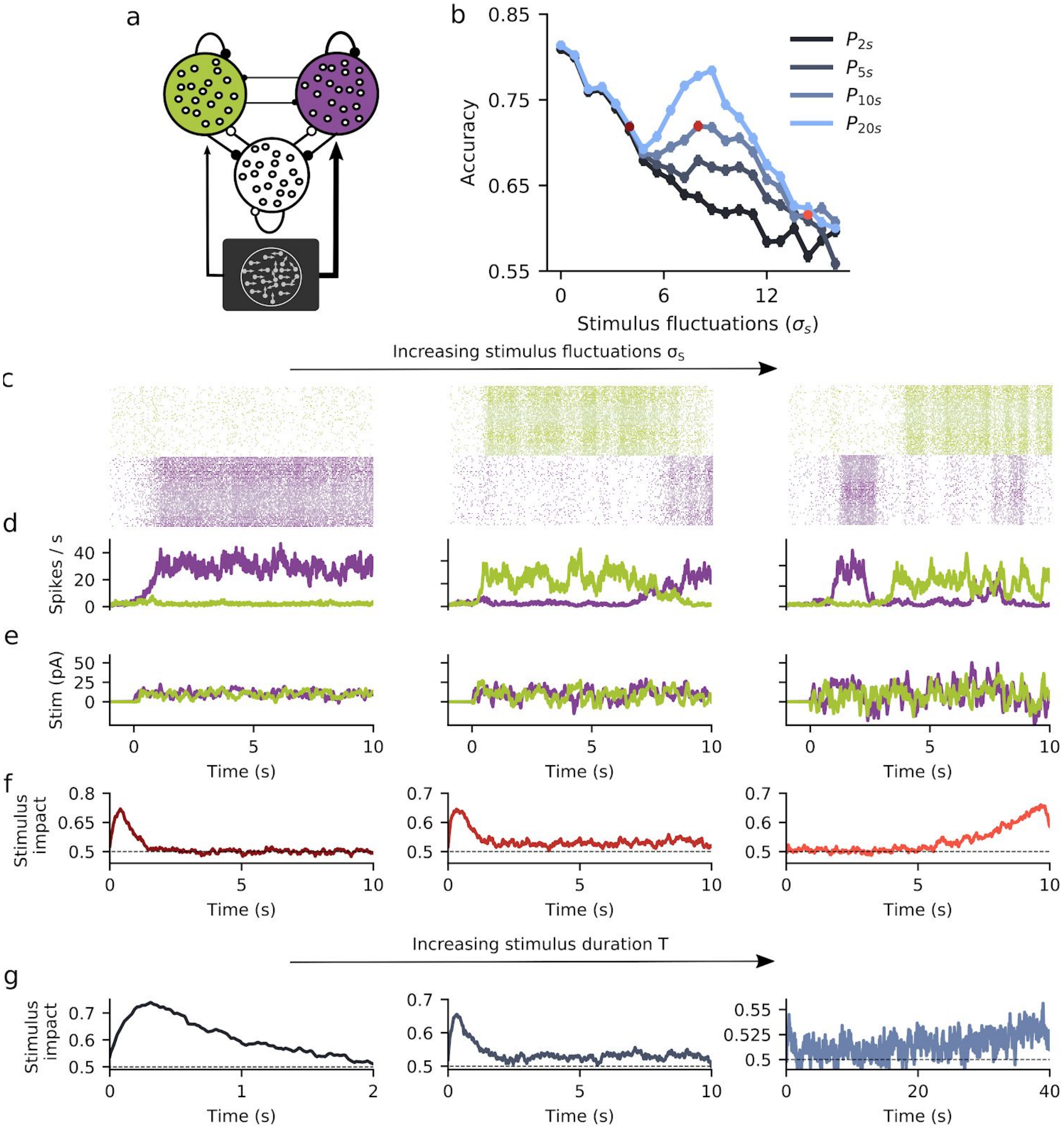

Supplementary Figure 4 | Flexible categorization dynamics in a spiking neural network model with AMPA, GABA and NMDA receptor dynamics.

To further test the generality of our results, we ran simulations with the classical leaky integrate-and-fire network model with conductance-based synapses by X.J. Wang [19]. This network has a similar architecture as our spiking neural network model (Methods) but includes more realistic synaptic interactions based on fast AMPA- and GABA-receptor dynamics (

$\tau_{AMPA} = 2\ ms$, $\tau_{GABA} = 5\ ms$) and slow NMDA-receptor dynamics ($\tau_{NMDA} = 100\ ms$). All model parameters were identical to the original publication[19], except for the stimulus input that was modeled as an input current as in the our model (Equation 45-46 in Methods; parameters: I_0 = 10 pA, μ = 0.08, τ_{stim} = 100 ms). The arrangement of the figure panels is identical to Fig. 5. (**a**) Schematic of the spiking network. (**b**) Accuracy P_C versus stimulus fluctuations σ_S obtained from simulations of the spiking network for four values of the stimulus duration T = 2, 5, 10 and 20 seconds (see inset). (**c-e**) Single trial examples showing the spike rastergram from the two excitatory decision populations (240 + 240 neurons) (c), traces of the instantaneous population rates (count window 30 ms) (d) and of the input stimuli (e), for different values of stimulus fluctuations $\sigma_S =$ 4 (left), 8 (middle) and 14 pA (right). Colored points in (b) indicate the σ_S used. (**f**) Psychophysical kernels obtained for each σ_S value. (**g**) Psychophysical kernels for $\sigma_S =$ 8 pA and different stimulus duration T = 2, 10 and 20 s, from left to right. In general, the results were qualitatively the same as for the spiking network with current-based synapses (Fig. 5) and the only change is that, due to the slower dynamics of the network, larger stimulus durations were required to reveal the non-monotonicity of the psychometric curve.

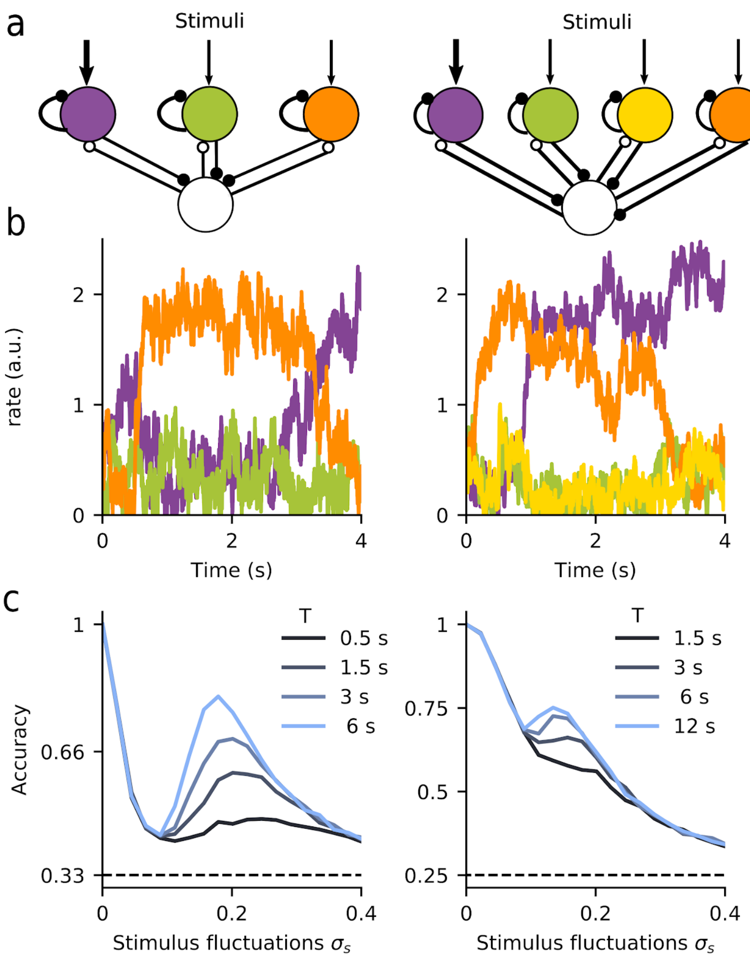

Supplementary Figure 5 | Flexible categorization dynamics in a multiple-choice task.

(**a**) Schematic of a *n*=3 (left) and *n*= 4 choice networks (right). Each network is composed of *n* excitatory populations (colored circles) each coding for a different choice, which compete via common inhibition (white circle). Fluctuating stimuli injected into each population are statistically identical except for one population which receives a higher mean input (see thicker arrow into purple population; see Methods for details). (**b**) Single trial examples showing the stochastic rate dynamics of the *n* excitatory populations. Both examples illustrate correcting transitions triggered by the stimulus fluctuations. The amplitude of the stimulus fluctuations was $\sigma_S = 0.18$ (left, *n*=3) and $\sigma_S = 0.13$ (right, *n*=4). (**c**) Accuracy $P(\sigma_S)$ versus the magnitude of the stimulus fluctuations σ_S obtained from numerical simulations of the rate-based network for four values of the stimulus duration (see inset). Dashed lines represent chance performance for n=3 (left) and n=4 choices (right).

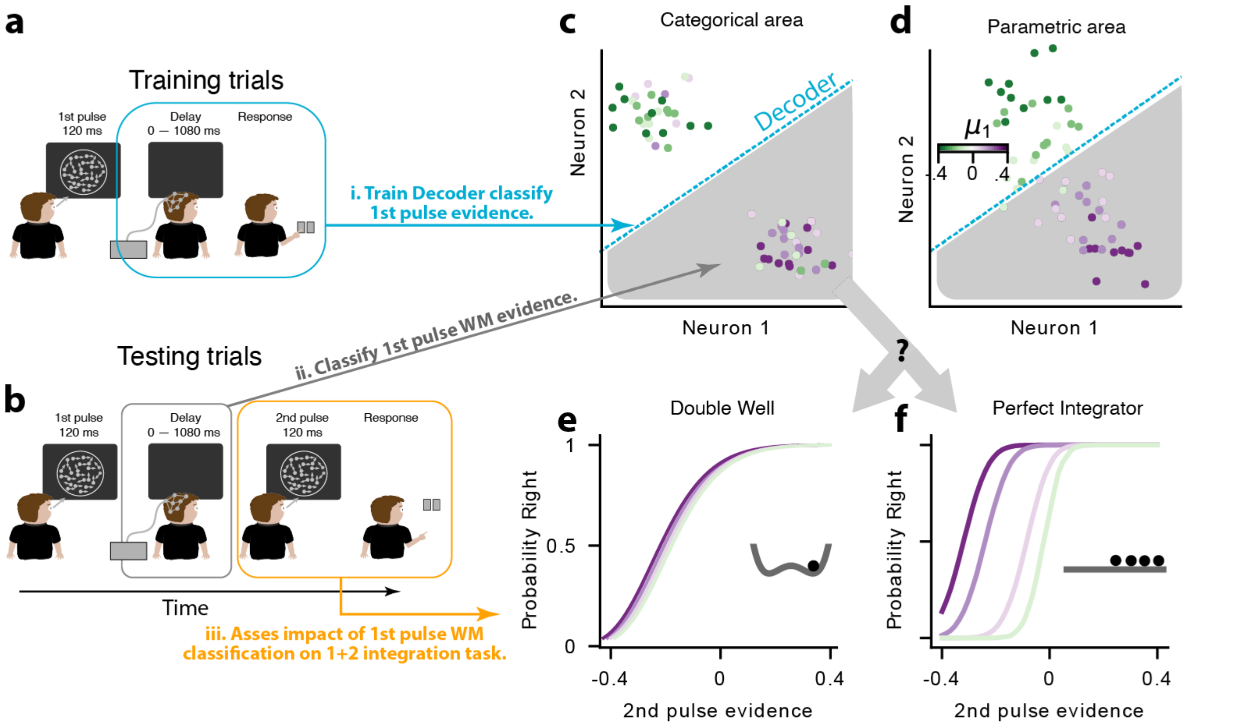

Supplementary Figure 6 | Categorical versus parametric working memory.

To further illustrate the implications of the double well model categorization dynamics in comparison to a perfect integrator we propose a modification of the two pulse experiment in which using neurophysiological recordings, the categorization of the 1st pulse evidence maintained during the delay period could be read-out. (**a-b**) The experiment consists of Training trials with a single pulse followed by a delay (a) and Testing trials with two pulses separated by the delay (b) as in the original task[35]. Training and Testing trials are interleaved during the experiment so that conditions are identical until the end of the delay. **i,** Recordings from the end of the delay period in Training trials are used to train a decoder (blue dashed line in c-d) to predict the categorical choice that is made in those single pulse trials. **ii,** Testing trials are then classified based on the categorization of the 1st pulse during the delay and to assess the impact of this classification on the final choice in these trials (e-f). **iii,** using only trials classified during the delay as e.g. Right (gray shaded areas in c-d) we use the second pulse evidence to plot psychometric curves about the final choice separately for each level of first pulse evidence μ_1 (see inset in d). (**c-d**) Categorization of the 1st pulse evidence stored during the delay in two different areas. Each dot represents the neural state of one trial at the end of the delay. The color of each dot is proportional to the coherence μ_1 of the first pulse (inset in d). The dashed blue lines are the category boundaries obtained from the decoder (panel a, step *i*). In a categorical brain area (c) evidence about μ_1 beyond the categorization is lost and there are only two clouds of dots representing left and right categories (this could be e.g. area FOF in[61]). In contrast, in a parametric area (d) the distance of each neural state to the category boundary carries information about μ_1 (this could be area PPC in[61]). Because different brain areas can

simultaneously encode the stimulus evidence with different degrees of categorization[61], one cannot conclude that the brain is using a categorical or a parametric representation to solve the task based on the neural representation alone. Instead, one needs to ask whether the category of the stimulus evidence read out during the delay is sufficient to describe the behavior in the double pulse trials (e) or whether in contrast, subjects modulate their responses beyond this categorical read out (f). **(e-f)** Probability of rightward choices vs. the mean evidence of the second pulse computed separately for each value of μ_1 using only trials categorized during the delay as Righward evidence (the same analysis can be done only using trials categorized as Leftward). For the double well model (e), conditioning on the 1st pulse category during the delay removes any dependence on μ_1 because all these trials start the integration of the second pulse in the right attractor (see inset). In contrast, for the perfect integrator (f), conditioning on the 1st pulse category does not remove the dependence on μ_1. Thus, our method should be independent of whether the recorded area stores the first stimulus evidence in a categorical manner (c) or in a parametric manner (d), because it is ultimately testing whether the categorization of this neural representation is or not sufficient to explain out the impact of the 1st pulse on choice.

Supplementary Methods

Simulations parameter of the double well model and canonical model.

Table 1

	μ	α	τ	σ_I	T	Bound
Figure 1	0	-	200 ms	0.1	1000 ms	0.5
Figure 2	0	1	200 ms	0.1	1000 ms	-
Figure 3 DWM	0.15	1	200 ms	0.0	2000 ms	-
Figure 3 DDMs	0.05	-	200 ms	0	2000 ms	0.5
Figure 4 DWM	0	1	200 ms	-	1000 ms	-
Figure 4 DDMs	0	-	200 ms	0.08	1000 ms	0.5
Figure 6	-	0.8	200 ms	0.3	-	-
Figure S1	0	-	200 ms	0.1	1000 ms	-
Figure S2	0	1	200 ms	0.1	-	-
Figure S3	-	1	200 ms	0.1	2000 ms	

Compatible parameters with a non-monotonic accuracy

Here we investigate the parameter range in which the accuracy is non-monotonic with the stimulus fluctuations. Concretely, we compute the critical values of the mean stimulus evidence (μ) and the internal noise (σ_I) beyond which the performance decays monotonically with the stimulus fluctuations σ_S. The attractor positions for weak stimulus strength μ are:

$$x_C = \sqrt{\frac{\alpha}{2}} + \frac{\mu}{4\alpha} + \mathcal{O}(\epsilon^2), \quad (1)$$

$$x_E = -\sqrt{\frac{\alpha}{2}} + \frac{\mu}{4\alpha} + \mathcal{O}(\epsilon^2) \text{ and} \quad (2)$$

$$x_U = -\frac{\mu}{4\alpha} + \mathcal{O}(\epsilon^2). \quad (3)$$

Plugging these into the transition rate equations, we obtain

$$k_C = \frac{2\alpha}{\sqrt{2}\pi} exp\left(-\frac{\alpha^2}{2\sigma^2} + \frac{2\mu}{\sigma^2}\sqrt{\frac{\alpha}{2}}\right) \text{ and} \quad (4)$$

$$k_E = \frac{2\alpha}{\sqrt{2}\pi} exp\left(-\frac{\alpha^2}{2\sigma^2} - \frac{2\mu}{\sigma^2}\sqrt{\frac{\alpha}{2}}\right). \quad (5)$$

We define the total level of noise (σ^2) as the sum of the stimulus fluctuations and the internal noise, ($\sigma^2 = \sigma_I^2 + \sigma_S^2$). If there is a non-monotonicity of the accuracy with σ, we should find a maximum of the probability of correct when the error attractor was first visited (p_C):

$$p_C = \frac{exp(a\beta)}{2cosh(a\beta)} \left(1 - exp(-kT)\right) \text{ with} \quad (6)$$

$$k = k_C + k_E = \frac{2\sqrt{2}\alpha}{\pi} exp\left(-\frac{\beta\alpha}{4}\right) cosh(a\beta), \quad (7)$$

Where $a = 2\mu\sqrt{\frac{\alpha}{2}}$ and $\beta = \frac{1}{\sigma^2}$. To check the existence of a local maximum we take the derivative of p_C with respect to σ

$$\frac{dp_C}{d\sigma} = -\frac{2}{\sigma^3}\frac{dp_C}{d\beta} = a\left(1 - tanh(a\beta)\right)\left(1 - exp(-kT)\right) + \frac{dk}{d\beta} T exp(-kT) = 0, \quad (8)$$

$$\frac{dk}{d\beta} = \frac{2\sqrt{2}\alpha}{\pi}\left(a sinh(a\beta) - \frac{\alpha^2}{2} cosh(a\beta)\right) exp\left(-\frac{\beta\alpha}{4}\right). \quad (9)$$

For small values of μ, the arguments of the trigonometric hyperbolic functions are very small and they can be approximated by $sinh(a\beta) \simeq 0$, $tanh(a\beta) \simeq 0$ and $cosh(a\beta) \simeq 1$. Using these approximations, equations 8 and 9 can be simplified as

$$\frac{dp_C}{d\sigma} = a\left(1 - exp(-kT)\right) + \frac{dk}{d\beta} T exp(-kT) \text{ with} \quad (10)$$

$$\frac{dk}{d\beta} = -\frac{\sqrt{2}}{\pi}\alpha exp\left(-\frac{\beta\alpha}{4}\right). \quad (11)$$

For small values of σ, $1 - e^{-kT} \simeq 0$. However there is always a large enough T so that $1 - e^{-kT} \simeq 1$. The local maximum of the accuracy must be in the region where these two effects are of the same order $kT \sim \theta(1)$ and the two terms in equation 10 cancel each other. Thus we defined $T = \frac{\tau}{\varepsilon}$ and $e^{-\frac{\beta\alpha^2}{2}} = \varepsilon y$, plugging these into equation 10, we obtain

$$\frac{dp_C}{d_\sigma} = a\left(1 - exp\left(-\frac{2\sqrt{2}\alpha}{\pi}y\tau\right)\right)\frac{\sqrt{2}y\tau\alpha^3}{\pi}exp\left(-\frac{2\sqrt{2}\alpha}{\pi}y\tau\right) = 0. \quad (12)$$

Let us define $z = \frac{2y\tau\alpha}{\pi}$. To have a maximum of p_C, there must be a solution to the following implicit equation

$$z = \frac{1}{\sqrt{2}}log\left(1 + \frac{\alpha\sqrt{\alpha}}{\mu}z\right). \quad (13)$$

Using the definitions of τ, y and z, we find a maximum of the probability of p_C as a function of the solution (z_0) of the implicit equation 13:

$$\sigma^2_{MAX} = \frac{\alpha^2}{2}\frac{1}{log\left(\frac{2T\alpha}{\pi z_0}\right)} \quad (14)$$

To find the maximum of the accuracy, we derive the probability of correct

$$P = P_0(1 - p_E) + (1 - P_0)p_C, \quad (15)$$

respect to σ :

$$\frac{dP}{d\sigma} = -\frac{2}{\sigma^3}\frac{d}{d\beta}\left(P_0 exp(-kT) + p_C\right), \quad (16)$$

where we rewrite equation 15 as a function of p_C and P_0. As long as $\frac{\sqrt{2\alpha}x_C}{\sigma} >> 1$, the probability to first visit the correct attractor,

$$P_0 = \frac{erf\left(\frac{\sqrt{2\alpha}}{\sigma}(x_0 + \frac{\mu}{2\alpha})\right) - erf\left(\frac{\sqrt{2\alpha}}{\sigma}(x_E + \frac{\mu}{2\alpha})\right)}{erf\left(\frac{\sqrt{2\alpha}}{\sigma}(x_E + \frac{\mu}{2\alpha})\right) - erf\left(\frac{\sqrt{2\alpha}}{\sigma}(x_C + \frac{\mu}{2\alpha})\right)} \quad (17)$$

is well approximated by

$$P_0 = \frac{1}{2}\left(1 + erf\left(\sqrt{2\alpha\beta}\left(x_0 + \frac{\mu}{2\alpha}\right)\right)\right) \quad (18)$$

In the range of parameters where μ is small and β is large we can assume that $\beta\mu^2 << 1$, $\sqrt{\beta\mu} << 1$ and $\mu\beta >> 1$. Using these inequalities and the definition of P_0 given in equation 18, we can simplify equation 16 to

$$\frac{dP}{d\sigma} = a\left(1 - tanh(a\beta)\right)\left(1 - exp(-kT)\right) + \frac{1}{2}\frac{dk}{d\beta}Texp(-kT) = 0. \quad (19)$$

With these simplifications, the derivative $\frac{dP}{d\sigma}$ is equivalent to the derivative $\frac{dp_C}{d\sigma}$ (equation 8) with a $\frac{1}{2}$ factor in the second term. This factor modifies the implicit equation 13 to

$$z = \frac{1}{\sqrt{2}}log\left(1 + \frac{\alpha\sqrt{\alpha}}{2\mu}z\right). \quad (20)$$

Then using the definition of z, the critical value of the internal noise for which accuracy decreases monotonically with the stimulus fluctuations is

$$\sigma_{IC}^2 = \frac{\alpha^2}{2}\frac{1}{log\left(\frac{2T\alpha}{\pi z_0}\right)} \quad (21)$$

where z_0 is a solution of the implicit equation 20. This implicit equation has two solutions, the trivial solution $z_0 = 0$ when σ is small and there are no transitions and a positive solution $z_0 > 0$. The accuracy is non-monotonic with the stimulus fluctuations when the positive solution exists. The positive solution of a general implicit equation of the form ($x = log(1 + cx)$) exists when the derivative of the right term at $x = 0$ is larger than 1, $(c > 1)$. In the case of equation 20, the positive solution exists when $\frac{\alpha}{2}\sqrt{\frac{\alpha}{2}}\frac{1}{\mu} > 1$. Thus there is a critical value of the mean evidence (μ) above which the accuracy decreases monotonically with the stimulus fluctuations

$$\mu_C = \frac{\alpha}{2}\sqrt{\frac{\alpha}{2}}. \quad (22)$$

Simulation parameters for the current-based spiking neural network model (Figure 5).

Table 2:

Populations		
N_E	1000	Size of excitatory populations A and B
N_I	500	Size of inhibitory population
Recurrent connectivity		
J_{EE}	0.16 mV	Weight of excitatory to excitatory connections
J_{IE}	0.08 mV	Weight of excitatory to inhibitory connections
J_{EI}	-4 mV	Weight of inhibitory to excitatory connections
C_{EE}	100	Average number of synaptic inputs from an excitatory population onto an excitatory neuron
C_{IE}	50	Average number of synapses for excitatory to inhibitory populations
C_{EI}	50	Average number of synapses for inhibitory to excitatory populations
Neuron model		
τ_m^E	20 ms	Membrane time constant of excitatory neurons
τ_m^I	10 ms	Membrane time constant of inhibitory neurons
g_L^E	12.5 nS	Leak conductance of excitatory neurons
g_L^I	25 nS	Leak conductance of inhibitory neurons
E_l	-70 mV	Resting potential
Θ	-50 mV	Spiking threshold
E_r	-60 mV	Reset potential
Synapse model		
τ_S^E	12.5 ms	Time constant of excitatory synapses
τ_S^I	1 ms	Time constant of inhibitory synapses
δ^E	5 ms	Mean synaptic delay for excitatory synapses (uniform distribution U(0, 10))
δ^I	1 ms	Mean synaptic delay for excitatory synapses (uniform distribution U(0,2))

External Poisson inputs		
J_{ext}	0.2 mV	Weight of external inputs
ν^{Eext}	5000 Hz	Firing rate of external Poisson inputs to excitatory neurons
ν^{Iext}	9000 Hz	Firing rate of external Poisson inputs to inhibitory neurons
Stimulus inputs		
I_0	25 pA	Mean input for zero-coherence stimulus
μ	0.015	Additional input for non-zero coherence stimulus
σ_S	varied	Amplitude of temporal modulations of the stimulus
τ_{stim}	20 ms	Correlation time of Ornstein-Uhlenbeck process
